# Syncope Caused by Huge Hiatal Hernia

**DOI:** 10.1155/2011/560734

**Published:** 2011-07-25

**Authors:** Gabriel Vanerio

**Affiliations:** Intensive Care Unit and Cardiology Departments, British Hospital Montevideo, 2420 Avenue Italia, 11600 Montevideo, Uruguay

## Abstract

A 84-year-old white female had a brief loss of consciousness while playing bridge. A few minutes before the episode she had eaten pizza and significant amount of carbonated soft drinks. After recovery, her friends noticed that she was alert, but pale and sweating. Upon arrival at the emergency room, sitting blood pressure was 160/60 mmHg with a normal sinus rhythm. A chest X-Ray was performed, which was essential to make the diagnosis. The X-Ray showed a large retrocardiac opacity with air and liquid level compatible with a giant hiatus hernia. After a copious snack the hiatal hernia compressed the left atrium, decreasing the left cardiac output, elucidating the mechanism of the syncopal episode. In patients presenting with swallow syncope (particularly after a copious meal, validating the importance of a careful history), a chest X-Ray should be always be performed.

Patients presenting to the emergency department with syncope are occasionally a diagnostic challenge. The cause of syncope might be revealed by a careful history and physical examination in approximately 40%–60% of patients. 

The chest X-Ray is a common test in patients presenting with syncope, but its value is less well-established. Unless guided by the history and physical examination findings, it is unlikely that a routine chest X-Ray will uncover the cause of syncope. We present a patient where the chest X-Ray illustrated the cause of syncope.

A 84-year-old white female with previous history of hypothyroidism and arterial hypertension had a brief loss of consciousness, while she was playing bridge. A few minutes before the episode she had consumed a significant amount of pizza and a carbonated soft drink. After recovery, her friends noticed that she was alert but pale and sweating. Upon arrival at the emergency room, she had a blood pressure of 160/60 mmHg with a normal sinus rhythm. The chest X-Ray (AP and lateral view) is shown below Figure 1(a). A large retrocardiac opacity is observed with air and liquid level compatible with a giant hiatus hernia. A CT-Scan with reconstruction, shown in the lower Figure 1(b), established the diagnosis. The mass is located behind the heart and in close relation with the left atrium. 

The cause of the syncopal episode might be related to the left atrial compression due to the sudden enlargement of the stomach. The patient underwent repair-reconstructive surgery (Nissen's fundoplication) with excellent result and no more syncopal episodes. 

Syncope is induced by various conditions. Swallow syncope, a vagally mediated reflex, constitutes a rare cause of syncope [1–6].

Some foods or beverages such as cold water, hot liquid, or carbonated drinks have been reported to trigger syncopal attacks. 

Despite swallow syncope caused by compression of the left atrium due to a huge hiatal hernia has been described previously [1–5], the chest X-Ray is the appropriate diagnostic test in this unusual type of syncope. 

We emphasize that a patient presenting to the emergency room with swallow syncope (particularly after an important amount of liquids), a chest X-Ray (AP and lateral views) should always be performed. 

In conclusion, we present a case of postprandial syncope in a patient with a large hiatal hernia that probably compressed the left atrium, thus causing an obstructive cardiac lesion with resultant syncope. 

## Figures and Tables

**Figure 1:**
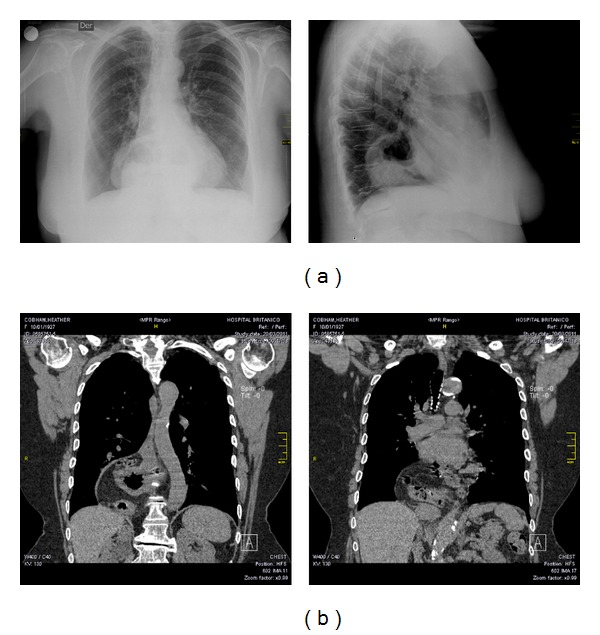
(a) A large retrocardiac opacity is observed with air and liquid level compatible with a giant hiatus hernia. (b) A CT-scan with reconstruction is shown validating the diagnosis. The mass corresponding to the stomach and likely small bowel is located behind the heart and in close relation to the left atrium.
